# Good‐bye to tropical alpine plant giants under warmer climates? Loss of range and genetic diversity in *Lobelia rhynchopetalum*

**DOI:** 10.1002/ece3.2603

**Published:** 2016-11-25

**Authors:** Desalegn Chala, Christian Brochmann, Achilleas Psomas, Dorothee Ehrich, Abel Gizaw, Catherine A. Masao, Vegar Bakkestuen, Niklaus E. Zimmermann

**Affiliations:** ^1^ Natural History MuseumUniversity of OsloOsloNorway; ^2^ Swiss Federal Research Institute WSLBirmensdorfSwitzerland; ^3^ Department of Arctic and Marine BiologyUiT – The Arctic University of NorwayTromsøNorway; ^4^ Institute of Resource AssessmentUniversity of Dar es SalaamDar es SalaamTanzania; ^5^ Norwegian Institute for Nature ResearchOsloNorway

**Keywords:** afro‐alpine, climate change, giant rosette plants, *Lobelia rhynchopetalum*, loss of genetic diversity, model algorithms, model complexity, range loss, tropical alpine plants

## Abstract

The main aim of this paper is to address consequences of climate warming on loss of habitat and genetic diversity in the enigmatic tropical alpine giant rosette plants using the Ethiopian endemic *Lobelia rhynchopetalum* as a model. We modeled the habitat suitability of *L*. *rhynchopetalum* and assessed how its range is affected under two climate models and four emission scenarios. We used three statistical algorithms calibrated to represent two different complexity levels of the response. We analyzed genetic diversity using amplified fragment length polymorphisms and assessed the impact of the projected range loss. Under all model and scenario combinations and consistent across algorithms and complexity levels, this afro‐alpine flagship species faces massive range reduction. Only 3.4% of its habitat seems to remain suitable on average by 2,080, resulting in loss of 82% (CI 75%–87%) of its genetic diversity. The remaining suitable habitat is projected to be fragmented among and reduced to four mountain peaks, further deteriorating the probability of long‐term sustainability of viable populations. Because of the similar morphological and physiological traits developed through convergent evolution by tropical alpine giant rosette plants in response to diurnal freeze‐thaw cycles, they most likely respond to climate change in a similar way as our study species. We conclude that specialized high‐alpine giant rosette plants, such as *L. rhynchopetalum*, are likely to face very high risk of extinction following climate warming.

## 1 Introduction

Both climate change and land use intensification following population pressure (Grünenfelder, 2005; Hedberg, 1964) are potential threats to high‐altitude species in tropical alpine habitats. The Intergovernmental Panel on Climate Change (IPCC, 2014) estimates that anthropogenically driven climate warming in the 21st century is likely to exceed 1.5°C relative to the 1850–1900 period in all scenarios and exceeds 2.0°C in many scenarios. The rate of temperature increase in mountainous systems is projected to be considerably higher, possibly two‐ to threefolds compared to that recorded during the 20th century (Nogués‐Bravo, Araújo, Errea, & Martinez‐Rica, 2007). As mountainous areas present steep biotic and abiotic gradients, such a dramatic change is expected to have a pronounced impact on biodiversity, ecosystems, and the services they provide (Price, 2000).

Organisms respond to changes in climate through adaptation (Davis, Shaw, & Etterson, 2005; Parmesan, 2006) or through range shifts toward the poles (Pearson, Dawson, Berry, & Harrison, 2002; Thomas, Franco, & Hill, 2006; Thomas et al., 2004) and higher elevations (Grabherr, Gottfried, & Pauli, 1994; Keller, Kienast, & Beniston, 2000; Lenoir, Gegout, Marquet, de Ruffray, & Brisse, 2008; Pauli et al., 2012; Pearson et al., 2002). Climate change is also expected to result in local or global extinction of many animals and plants (Dullinger et al., 2012; Thomas et al., 2004, 2006; Thuiller, Lavorel, Araujo, Sykes, & Prentice, 2005). Species that are confined to marginal areas such as mountain tops, polar regions, and islands may not have sufficient suitable habitat left to escape climate change and hence may face massive range contraction associated with high risks of extinction (Dullinger et al., 2012; Lenoir et al., 2008).

Upward shifts in response to recent climate change are already measurable, although they tend to lag behind the velocity of climate change (Bertrand et al., 2011). Several historical surveys using permanent plots have shown that species with high thermal demands are invading higher zones, whereas species with lower thermal requirements are retreating to even higher altitudes (Keller et al., 2000; Lenoir et al., 2008; Pauli et al., 2012). A study of 26 peaks exceeding 3,000 m in Western Austria and Eastern Switzerland found up to 70% increase in species richness since the beginning of the 20th century due to immigration of lower‐elevation species (Grabherr et al., 1994). A comparison of the altitudinal distributions of 171 forest species between 1905 and 2005 showed, on the average, 29 m upward shift of the species’ distribution centers per decade (Lenoir et al., 2008). Nearly the same average upward shift rate (27 m per decade) was reported for 125 species endemic to the Himalaya Mountains (Telwala, Brook, Manish, & Pandit, 2013). An analysis of a long‐term data set on the upper limits of plants from the beginning of the 20th century for 25 mountains in the European Alps showed an upward shift exceeding 100 m for most species, with an average of about 10 m per decade, and 33 species were recorded as new to the summits (Frei, Bodin, & Walther, 2010). The nival species of Swiss and Austrian peaks were reported to migrate 5 m upwards per decade (Grabherr et al., 1994).

There are also conceptual (Lenoir et al., 2010) and observation‐based reports (Lenoir et al., 2008; Pauli et al., 2012) indicating that a considerable proportion of species does not follow this general trend, with many species not moving, or even moving their centers of elevation distribution downslope. Habitat modification, changes in rain and snowfall regimes, and changing competitive interactions among species are thought to explain these downward range shifts (Lenoir et al., 2010). In Mediterranean mountains, species richness has been found to decrease probably due to decreased moisture availability (Pauli et al., 2012). This illustrates that the effects of changing temperatures are not always easy to project, as climate change may have cascading effects on abiotic and biotic factors.

One of the most important features characterizing tropical alpine habitats is the lack of pronounced seasonal variation in temperature. Instead, the diurnal variation in temperature is extremely high with warm days alternating with very cold nights, when temperatures can drop well below 0°C. Tropical alpine habitats are therefore unique, and they are famous for their conspicuous giant rosette plants, which show intricate adaptations to such extreme diurnal temperature variations (Fetene, Gashaw, Nauke, & Beck, 1998; Halloy, 1983; Hedberg, 1964; Nagy & Grabherr, 2009). Because of prolonged cold stress and snow accumulation in winter, temperate alpine habitats do not sustain plants of such large life forms, while the highest mountains in tropical Africa are renowned for their many, and often vicariant, species of giant groundsels (*Dendrosenecio*) and giant lobelias (*Lobelia;* Hedberg, 1964).

Although many giant rosette plants are only distantly related phylogenetically, they have developed similar distinctive morphological, physiological, and life history adaptations to the diurnal freeze‐thaw cycles of tropical alpine habitats (Halloy, 1983). These complex adaptations include the following: (i) retaining old leaves as a shelter, (ii) accumulating water within body parts to minimize thermal shocks, and (iii) having large rosette leaves which fold during the freezing nights through nyctinastic leaf movements to protect the delicate buds. They also share some seemingly paradoxical morphological and physiological traits. In tropical as well as other mountains, the height of plants normally decreases with increasing altitude (Smith, 1980), while giant rosette plants have evolved a noticeable exception to this general pattern as one of their adaptation mechanisms (Fetene et al., 1998; Meinzer, Goldstein, & Rundel, 1985; Smith, 1980; Smith & Young, 1987). The temperature near the ground is very low during night and highly fluctuating throughout the day as compared to the temperature more than 1 m above the ground (Fetene et al., 1998). The main advantage gained by a tall life form is to escape the strong diurnal temperature fluctuation near the ground and to avoid freezing of apical meristems (Fetene et al., 1998). As the freeze‐thaw cycles aggravate with increasing altitude, giant rosette plants grow taller at higher altitudes in order to keep the apical meristem farther away from the ground where freezing is more severe. Taller plants have faster growth rate, implying that their growth rate increases with age, representing yet another paradox. In addition, the amount of water stored in their pith and leaves also increases with altitude (Smith & Young, 1987).

In this study, we assess the risk of losing habitat and genetic diversity in the iconic giant rosette plant *Lobelia rhynchopetalum* Hemsl. due to climate change in the Ethiopian mountains, which represent the largest contiguous high‐mountain system of Africa. Under the current climate, this species is widespread and reaches the highest peaks in the country, such as in the Simen and Bale Mountains and in Mount Choke, and it can therefore be expected to lose its range under future warmer climates. *Lobelia rhynchopetalum* is endemic to the Ethiopian mountains and the only representative of the giant rosette life form in this high‐alpine area. While most other plants rarely exceed 0.5 m in height in the alpine zone of Ethiopia, *L. rhynchopetalum* can grow taller than 10 m (Fetene et al., 1998). It is confined to the alpine zone and thus serves as a conspicuous indicator species of the tropical alpine habitat. In addition, it is a tourist attraction and it has thus not only ecological importance but also recreational and economic value (Geleta & Bryngelsson, 2009).

As *L. rhynchopetalum* has developed adaptations to the high‐alpine tropical climate similar to those of other tropical alpine giant rosette plants through convergent evolution, it can be considered as representative of other giant rosette plants with respect to vulnerability to climate change. Here, we project the current distribution of *L. rhynchopetalum* and evaluate how its habitat range may respond to different future climate scenarios. We do so by using an ensemble approach using three different statistical methods, and by fitting both a simple and a complex model parameterization (Merow et al., 2014) for each model for building the ensemble. We also assessed its current genetic diversity by genotyping samples from three main mountain systems across the Ethiopian Highlands (Simen, Bale, and Choke) using genome‐wide amplified fragment length polymorphism (AFLP) markers. In particular, we estimate to what degree genetic diversity is geographically structured in these archipelago‐like African high mountains and predict loss of genetic diversity due to climate‐driven range loss.

## 2 Materials and methods

### 2.1 Study species and distribution data

*Lobelia* (Lobeliaceae) comprises ca. 300 mainly tropical/subtropical species, of which 18 occur in Ethiopia (Thulin, 2006). The exclusively alpine *L. rhynchopetalum* (ca. 3,400–4,500 m) is characterized by a single erect stem growing to 10 m or more in height, by entire, smooth, glossy, lanceolate to oblanceolate rosette leaves up to 80 cm long and 12 cm wide, and by a single, dense, and several meters long terminal inflorescence.

**Figure 1:**
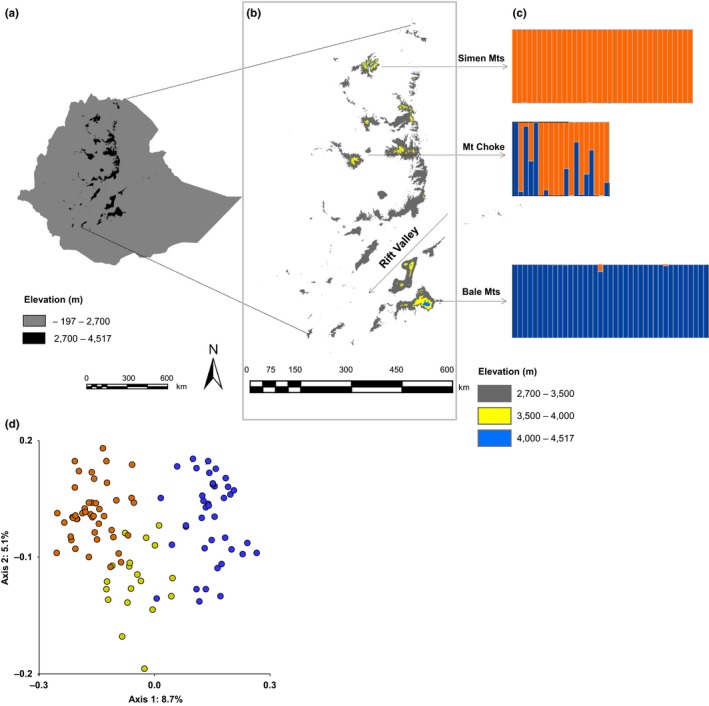
Study area and geographic structuring of genetic variation in *Lobelia rhynchopetalum*. (a) Ethiopia; (b) the Ethiopian high landmasses (>2,700 m a.s.l.) considered for habitat suitability modeling and the three mountain systems investigated in the field; (c) two genetic groups (blue and orange) inferred from Bayesian clustering of amplified fragment length polymorphism (AFLP) genotypes; and (d) Ordination (PCoA) of AFLP genotypes showing geographical structuring (blue: Bale Mts, yellow: Mt Choke, orange: Simen Mts)

For sampling, we selected three Ethiopian alpine mountain systems located at different latitudes. The Bale Mountains (6.9^o^N, 39.7^o^E) are found southeast of the Great East African Rift Valley, while Mount Choke (10.7^o^N, 37.8^o^E) and the Simen Mountains (13.2^o^N, 38.8^o^E) are located northwest of the Rift Valley (Figs. 1a, b). We sampled a total of 24 transects running downwards in eight different cardinal directions from mountain peaks (starting as high as 4,500 m a.s.l.) that were found on different sides of each mountain system to below the treeline (ca. 3,500 m). At each 50 m altitudinal interval in each transect, the presence or absence of *L. rhynchopetalum* in one 20 m x 20 m plot was recorded (Table S1). For the area below the treeline, where *L. rhynchopetalum* is known to be absent, we generated additional, random pseudo‐absence points in a number proportional to the area‐weighted observation density made above the treeline. These two data sets were combined and used for model training. In total, we sampled 218 plots (87 presences), and we added 612 pseudo‐absences from below the treeline. For model evaluation, we used an independent presence–absence data set obtained as part of separate field campaigns in the same three mountain systems (for collection of samples for the genetic diversity study, see below). This data set contained 26 presence and seven absence points to which we added 113 pseudo‐absence points below the treeline using the same area‐based weighting.

### 2.2 Predictor variables

Averages of monthly precipitation sum and minimum and maximum monthly temperature at 1 km spatial resolution of both current and future climate using different scenarios were obtained from the global climate data set WorldClim (Hijmans, Cameron, Parra, Jones, & Jarvis, 2005). In order to better characterize the complex mountain topography on the basis of these climate maps, we downscaled the temperature variables to 270‐m spatial resolution following the procedure of Zimmermann, Edwards, Moisen, Frescino, and Blackard (2007), and we interpolated the precipitation layers to the same resolution. We computed 19 bioclimatic variables according to Hijmans et al. (2005), resampled the global 90 m resolution SRTM DEM (Jarvis, Reuter, Nelson, & Guevara, 2008) to 270 m resolution, and derived maps of slope angle, slope aspect, and topographic position index (TPI; Jenness, 2006). We used eight categorical classes for aspect (N, NE, E, SE, S, SW, W, NW). To obtain the appropriate scale at which TPI explained the variation in the data set, we produced six different TPI maps (tpi3–tpi8) by setting the neighbor shape to rectangle and by varying the number of neighborhood cells from 3 × 3 (for tpi3) to 8 × 8 (for tpi8) in ArcGIS using Land Facet Corridor Tools (Jenness, Brost, & Beier, 2013) and compared their performance in MaxEnt (Phillips, Anderson, & Schapire, 2006). We used GlobCover (Arino et al., 2012) by clipping it to the study area boundary and resampling it to 270 m using nearest neighbor method to represent a categorical landcover variable. Finally, we overlaid these rasters with the presence and absence points of both plot data sets. For further analyses, we first calculated Pearson's correlations between predictor variables and retained only one of those with correlations ⋝0.7, selecting the one with highest assumed biological importance (Fig. S1). We finally kept ten variables for model building: slope angle, slope aspect, TPI7, landcover, mean diurnal range of temperature (BIO2), maximum temperature of the warmest month (BIO5), annual temperature range (BIO7), annual precipitation (BIO12), precipitation of the warmest month (BIO13), and precipitation of the coldest quarter (BIO19).

### 2.3 Model fitting and evaluation

For mapping potentially suitable habitats for *L. rhynchopetalum*, we selected three model algorithms that differ in general structure and statistical properties: (i) MaxEnt, based on parametric maximum likelihood (Halvorsen, 2013; Halvorsen, Mazzoni, Bryn, & Bakkestuen, 2014; Phillips et al., 2006); (ii) GAM, based on nonparametric maximum‐likelihood functions (Wood, 2011); and (iii) GBM, based on resampling (boosting) methods (Friedman, Hastie, & Tibshirani, 2000). For each model algorithm, we implemented two model versions, one generating simple and one generating complex response shapes. We finally built ensemble maps of current and projected future ranges of the species. The three model algorithms differ in how they relate a response to predictors, and the two model complexity levels differ in the degree to which they fit the response to the data (Merow et al., 2014). In all three models, we weighted the presences and absences inversely proportional to their fraction, because we had ca. 3× more absences than presences available in both the training and evaluation data set.

For GAM, we used the mgcv package (Wood, 2011). In order to generate shapes of varying complexity, we set the k parameter in the spline smoother to k = 3 for simple and to k = 8 (i.e., the largest possible allowed by the model) for the complex model. For both model complexity settings, a manual predictor variable selection was carried out by stepwise dropping the least significant variable until the two consecutive models revealed a significant difference in the variation they explained at a 95% confidence level in an ANOVA chi‐square test.

For GBM, the simple response was generated by setting the tree complexity parameter to two in the “gbm.step” function of the DISMO package (Hijmans, Phillips, Leathwick, & Elith, 2013) and “n.drops” was set to “auto” from the “gbm.simplify” function in the gbm package (Ridgeway, 2013). No interaction among variables is thus allowed in this setting. For the complex response, we set the tree complexity to five and “n.drops” to zero, which allowed for significant (and more complex) interactions among variables during model building. For both complexity versions, several models were run by alternatively setting the bag fractions to 0.5 and default (i.e., 0.75; Elith, Leathwick, & Hastie, 2008) and using different values of learning rates (slightly varying values ranging from 0.0001 to 0.05). For final model runs, we used those combinations of bag fractions and learning rates for which runs of more than 1,000 trees (see Elith et al., 2008) yielded lowest cross‐validation deviances.

For MaxEnt, different penalty terms with regard to values of the regularization multiplier were used to regulate model complexity and overfitting. We built several models starting with a low penalty term of 0.5 and slightly increased the penalty term until smooth response curves were obtained (penalty term of 8 in our case) for most of the continuous predictor variables. Thus, we used a regularization multiplier of 8 for the simple, while we used a low penalty term with a regularization multiplier value of 0.5 for the complex model version.

All models were validated against the independent data set using AUC as accuracy metric. We used three different criteria for splitting the probabilistic predictions into binary presence–absence maps for each model: (i) maximum sum threshold (MST), the probability value at which the sum of sensitivity and specificity is maximized; (ii) prevalence threshold (PT), with the prevalence as probability threshold; and (iii) maximum Kappa (MK), the threshold that provides the highest kappa value. By this, we generated six binary maps per model algorithm (18 in total). Finally, we combined all 18 binary maps and classified them into three habitat ensemble suitability classes based on the proportion of models that map the presence of the species per pixel: (i) <30% predict the presence (=unsuitable habitat with high certainty), (ii) 30%–60% predict the presence (=uncertain habitat suitability), and (iii) >60% predict the presence (=suitable habitat with high certainty).

Using two different future climate models, the community climate model version 3 (CCMA3) and the Hadley Center Coupled Model version 3 (HadCM3), and two emission scenarios (A2 and B2) for the year 2080, we estimated the range of the species (where >60% agreement for suitable habitat in model ensembles was obtained) and carried out range change detection. Future projection ensembles were thus built on 72 maps, as each model, version, and threshold were applied to 2 GCMs and two scenarios, each.

### 2.4 Genetic analyses

Leaf material of *L. rhynchopetalum* was obtained from the same three mountain systems (Figure 1a,b) and dried in silica gel. Leaves from five individual plants were collected within a 100 m × 100 m plot to represent a local population. A total of 102 individuals from 21 populations were successfully analyzed for AFLPs (see Appendix S1 for details). To quantify genetic diversity, we estimated the proportion of polymorphic markers (*P*), Nei's gene diversity (*D*), and genetic rarity (*Ra*) using AFLPdat (Ehrich, 2006). We also counted the number of AFLP markers that were private to each mountain system and the number of markers that were shared among mountain systems. Genetic structure was assessed using principal coordinate analysis (PCoA) based on Dice's similarity coefficient and a Bayesian model‐based clustering method. Levels of genetic differentiation were estimated in an analysis of molecular variance (AMOVA; Appendix S1).

We quantified the expected loss of genetic diversity due to range loss using a similar approach as Alsos et al. (2012). As genetic data were not available from the whole range of the species, in particular not from some of the areas that remained suitable under projected climate change (e.g., Mt Guna, Shewa‐Wallo), loss of genetic diversity was estimated by randomly removing a proportion of sampling localities proportional to the area projected to be lost. Genetic diversity in the remaining populations was expressed as the number of remaining markers, as this diversity measure is more sensitive to rapid decrease in population size than Nei's gene diversity (El Mousadi & Petit, 1996). Assuming that a complete loss of gene diversity (i.e., all remaining individuals identical) would correspond to the average number of markers per individual, the proportion of genetic diversity expected to be lost was calculated as 1 − (*N*_r_ − *N*_tot_)/(*N*_poly_ − *N*_tot_), where *N*_r_ is the number of markers in the remaining populations, *N*_tot_ is the average number of markers per individual, and *N*_poly_ is the total number of polymorphic markers (Alsos et al., 2012). The random removal procedure was repeated 200 times and reported as average predicted loss with 95% confidence intervals (CI). As genetic data were not available from the whole range of the species, in particular not from some of the areas that remained suitable under projected climate change (e.g., Mt Guna, Shewa‐Wallo), loss of genetic diversity was estimated by randomly removing a proportion of sampling localities proportional to the area projected to be lost. We thus assume that the genetic loss predicted in response to the area loss is proportional to the loss that can be expected for range‐wide genetic diversity. To strengthen our assumption, we have also repeated this analysis by constraining the surviving population to different mountains, so that the loss is additionally distributed evenly among mountains.

## 3 Results

### 3.1 Habitat suitability modeling

All the three statistical models generated similarly high AUC values against the independent data set (0.945–0.973; Table S2). GBM performed best at both complexity levels followed by MaxEnt and GAM. The AUC difference between complexity levels was low. Interestingly, the simple models performed slightly better than the more complex ones when applied to the independent test data set. The predicted distribution patterns obtained from all models and both complexity levels were also quite similar (Fig. S2). However, the binary presence–absence maps produced with different methods for splitting the probability predictions generated quite different results, with the PT producing much smaller suitable habitats and thus contributing a considerable amount of uncertainty to the map products (Figs. S3–S8).

**Figure 2:**
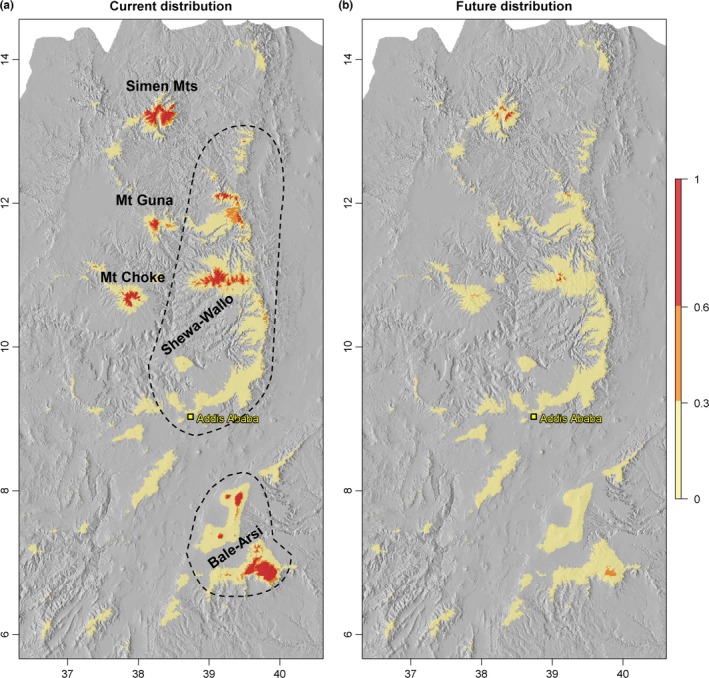
Maps representing the ensemble mean of suitable habitat for *Lobelia rhynchopetalum* produced by combining (a) 18 binary presence–absence maps (from three model algorithms at two complexity levels and three probability thresholds; see Figs. S3–S8) showing the current suitable habitat and (b) 72 binary presence–absence maps (from three algorithms with two complexity levels and three probability thresholds for each of the 2 GCMs and two emission scenarios) showing the remaining suitable habitat by 2,080. 0.0–0.3 unsuitable with high certainty, 0.3–0.6 uncertain, >0.6 suitable with high certainty

The binary ensemble maps for each method and complexity level (Fig. S9) and the final binary ensemble maps combining all models and complexity levels (Figure 2a) consistently indicated that most of the alpine regions in Ethiopia currently presents suitable habitat for *L. rhynchopetalum*. The species has its largest zone of suitable habitat south of the Rift Valley in the Bale‐Arsi region (Table 1; Figure 2a). The second largest suitable area is found at the northwestern edge of the Rift (Shewa‐Wallo‐Guna massif) followed by the Simen Mountains. However, the projections under both emission scenarios for both climate models suggested that *L. rhynchopetalum* would lose a large portion of its current habitat and would be confined to areas north of the Rift Valley, primarily to the Simen Mountains (Figure 2b; Figs. S10–S13). The climate model HadCM3 projects higher warming and thus constrains the suitable habitat under future climate scenario even more severely (Table S3).

**Table 1:** Predicted habitat suitability for *Lobelia** **rhynchopetalum* in different mountain regions

Mountain regions	Current habitat range (km^2^)	Predicted to remain suitable (%)	Predicted to be lost (%)	Predicted to be uncertain (%)
Bale‐Arsi Mts	1,122.0	0.0	87.0	13.0
Mt Choke	259.3	0.0	91.0	9.0
Simen Mts	606.9	12.6	68.9	18.4
Shewa‐Wallo‐Guna Mts	765.8	2.4	84.2	13.4
Total	2,754	3.4	82.6	13.9

**Figure 3:**
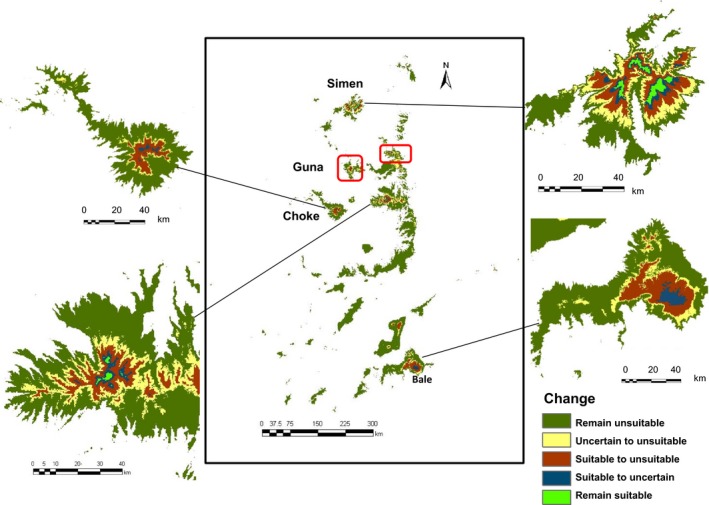
Overlay of the current and future habitat suitability maps showing predicted range loss for *Lobelia rhynchopetalum*. Details are shown for the three mountain ranges from where samples were collected and one of the mountains on which the remaining suitable habitat is predicted to occur with high certainty in 2,080. The two other mountains tops in which the habitat of *L. rhyncopetalum* is predicted to remain suitable are indicated in red rectangles

Overlaying projected future with current habitat suitability maps revealed that no habitat remained suitable with certainty in two of our study areas, Bale Mts and Mt. Choke (Figure 3, Table 1). In the third sampling area, in Simen Mts, only 13% of the currently suitable habitat predicted to remain suitable with certainty. In the fourth area, the Shewa‐Wallo‐Guna massif, where no samples were collected for genetic analysis, the current habitat that would remain suitable under future climates with certainty is only 2.4%. Over Ethiopia as a whole, only 3.4% (93.6 km^2^) of the current habitat would remain suitable, and the comparably large and disconnected current range of the species would be further fragmented and confined to four mountaintops (Figure 3). Another 13.9% of the current range would present uncertain habitat suitability due to differences among climate and statistical models, complexity levels and thresholds (Table 1), which translated to a total of 82.6%–96.6% range loss for *L. rhynchopetalum*. Currently, *L. rhynchopetalum* was predicted to occur between 3,300 m and the highest peaks (4,500 m; Fig. S14), whereas under future climates, the lower altitudinal limit would shift upward to 3,950 m. Similarly, the optimum altitude would shift from about 3,700 to 4,100 m.

### 3.2 Genetic data

The final genetic data matrix consisted of 102 individuals genotyped for 173 polymorphic markers. The genetic diversity estimates varied little among the three mountain systems, although there were somewhat fewer polymorphic loci and private markers in Choke than in the two other massifs (Table 2). Of the 173 polymorphic markers, only 63 (36.84%) markers were shared among the three mountains while 41 (23.69%) markers were not found in the samples from Simen Mts where we expect the largest portion of the suitable habitat under future climate scenario (Fig. S15). The PCoA revealed a continuous gradient in genetic variation ranging from Bale via Choke to Simen plants along the first axis, which explained only 8.7% of the variation (Figure 1d). The Bayesian clustering analysis suggested a similar structure with two genetic groups: one contained all plants from Bale and the other contained all plants from Simen, whereas both genetic groups were represented in Choke (Figure 1c, S16). In the AMOVA, most (80%) of the variation was found within populations, but significant albeit moderate variation was also found among mountain systems (12%) and among populations within mountain systems (8%; Table S4).

**Table 2:** Genetic (AFLP) diversity in *Lobelia rhynchopetalum* estimated in total per mountain system and as average of population estimates

Mountain system	*N*/pop	Mountain system in total			Average of population estimates	
		*P*	*D*	Private	D_AV_ [min–max]	Ra [min–max]
Bale Mts	39/8	73.41	0.127	19	0.114 ± 0.02 [0.089–0.139]	1.737 ± 0.441 [1.205–2.394]
Mt Choke	19/4	53.18	0.123	5	0.116 ± 0.01 [0.108–0.120]	1.766 ± 0.29 [1.341–1.982]
Simen Mts	44/9	72.83	0.128	21	0.120 ± 0.02 [0.092–0.154]	1.666 ± 0.446 [1.092–2.402]

*N*, number of individuals; pop, number of populations; *P*, percentage of polymorphic markers; *D*, Nei's gene diversity; private, number of private markers; D_AV_, average Nei's gene diversity per population; Ra, average genetic rarity.

**Figure 4:**
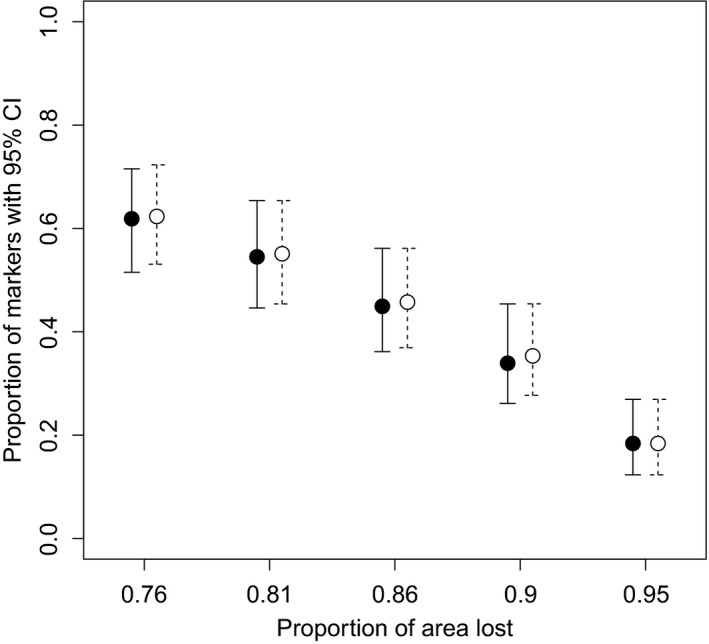
Predicted loss of genetic diversity, expressed as the proportion of polymorphic markers persisting, in *Lobelia rhynchopetalum* as a result of range loss by 2,080. Black dots represent mean estimates for random survival of populations, and white dots represent mean estimates obtained by assuming that populations survive in at least two mountain systems (with 95% CI in both cases)

Removal of 16–20 of the 21 analyzed populations in 200 random repeats, corresponding to a range loss of 76%–95%, resulted in loss of 37.4%–81.6% of the AFLP markers (Figure 4). The predicted loss of genetic diversity was largely proportional to the range reduction and decreased rapidly with higher numbers of surviving populations. As our range modeling projected that *L. rhynchopetalum* may survive in four different mountain systems, the reduction in genetic diversity was also estimated forcing remaining populations to be on different mountain systems. These results were almost identical to those from the random resampling (Figure 4; Table S6–S7).

## 4 Discussion

### 4.1 Impact of climate change on habitat range

In this study, we predicted that almost all Ethiopian alpine areas are suitable for *L. rhynchopetalum* under the current climate. This is consistent with the Flora of Ethiopia and Eritrea (Thulin, 2006), where the species is reported from most floristic regions in Ethiopia. Under warmer climates, however, we found that this enigmatic alpine giant will suffer massive reduction in range. Today, the species has its largest proportion of suitable habitat south of the Rift Valley, that is, in the Bale‐Arsi massifs. Under all emission scenarios and model algorithms considered, we predicted that no habitat will remain suitable with high certainty in any of the mountain ranges south of the Rift by the end of the 21st century, indicating very high risk of local extinction. Furthermore, no habitat in Mt Choke is projected to remain suitable with high certainty. Under future climates, we found that suitable habitat for *L. rhynchopetalum* will be confined to only four mountaintops in two areas northwest of the Rift Valley, the Simen Mts and the Shewa‐Wallo‐Guna massif. Thus, *L. rhynchopetalum* will lose its range at low latitudes as well as low altitudes.

Such large range reductions are typical of mountain systems that provide little room for upward movement to higher altitudes. In a study of climate change impacts on European mountain plants, similar range losses as we found here were projected for the Pyrenees and the far eastern part of the European Alps, while species in most other systems, such as the main parts of the Alps, the Carpathians, and the Scottish and Scandinavian mountain ranges are projected to be less threatened by climate change (Engler et al., 2011). Also, projected range loss in lowlands is usually much smaller than that projected for mountaintops that barely reach above treeline (Lenoir et al., 2008; Thomas et al., 2004, 2006; Thuiller et al., 2005). Tropical mountains are specifically threatened to such strong range reduction effects due to both the comparably strong fragmentation of the disconnected mountains and the comparably little reach above treelines in most of these mountains, which represent islands in a sea of lowland tropical vegetation.

Our results for *L. rhynchopetalum* add to a growing body of evidence showing dramatically high rates of range loss and altitudinal shifts in mountain plants (e.g., 27 m in altitude per decade for Himalayan endemics; Telwala et al., 2013). Such high rates can be expected because of the enhanced changes anticipated in climates in mountainous regions (Nogués‐Bravo et al., 2007), typically narrow habitat ranges and low genetic diversity that may counteract local adaptation in high‐alpine taxa, and steep biotic and abiotic gradients in alpine areas. In *L. rhynchopetalum*, the habitat predicted to remain suitable with high certainty is very small in spatial extent, only 936.4 km^2^. Furthermore, this small area seems to be fragmented among four mountaintops, which may be too small to sustain viable populations (Figure 3). Thus, in terms of range loss and fragmentation alone, not considering the expected dramatic genetic erosion in the species (see below), *L. rhynchopetalum* can be considered to face high risk of extinction and the Ethiopian alpine region may lose its only representative of the iconic tropical alpine plant giants. Other tropical alpine giant rosette plants will likely be affected in a similar way by a warmer climate, as they occupy the uppermost alpine habitats with narrow ecological amplitudes and possess similar morphological and physiological syndromes developed in response to the diurnal freeze‐thaw cycles through convergent evolution. Further adding to the threat against the tropical alpine giants is the expected upward shift of the treeline and invasion of new species from lower vegetation belts, resulting in shrinking of the alpine habitat and increased competition. Thus, we might lose these high‐alpine, charismatic flagship species that render the tropical alpine areas so peculiar.

### 4.2 Impact of climate change on genetic diversity

Tropical African alpine habitats represent highly fragmented ecosystems being restricted to the mountain peaks that rise above 3,500 m resulting in geographic isolation among populations (Hedberg, 1951). In *L. rhynchopetalum*, we found significant albeit moderate geographic structuring in its genetic diversity, corresponding to the three major mountain systems analyzed. This is consistent with the statistically significant variation among populations of *L. rhychopetalum* from Simen and Bale reported based on sequences of the nuclear ITS region and eight plastid DNA regions (Geleta & Bryngelsson, 2009). In our study, we found considerable numbers of AFLP markers confined to a single mountain system. Only 36.4% of the markers were shared among the three mountain systems, and 23.9% of the total markers were absent from Simen Mts, where we expect the largest patch of suitable habitat to remain under future climates (Fig. S15). Our results clearly show the adverse genetic consequences of habitat loss from the two sampling areas in particular and in other geographic regions in general.

The overall projected habitat loss ranges from 82.6 to 96.6%, with only 3.4% of the habitat being projected to remain suitable with high certainty. Randomly retaining sample populations equivalent in number with the habitat that remains suitable with high certainty and re‐computing the genetic diversity indicated an average loss of 82% of the AFLP markers, which is almost as high as the range loss (Figure 4). However, range loss due to climate change is not random. It mainly occurs at the organisms’ lower elevational and latitudinal range edges. Although selection pressures may vary in different parts of the range, we gave equal weight to all loci as typical in studies relying on AFLP markers, of which the majority represents neutral genetic diversity (Alsos et al., 2012). Range loss from different areas is likely to have a similar impact on genetic diversity when genetic diversity is distributed evenly across geographic regions (Alsos et al., 2012). We found similar levels of genetic diversity in *L. rhyncopetalum* populations from different mountains, and constraining the surviving populations to different mountaintops resulted only in small differences in genetic diversity loss compared to randomly sampling the surviving populations.

In a compilation of 307 studies of plant species, Nybom (2004) found an average AFLP‐based genetic diversity of 0.23 ± 0.08, with highest diversity in long‐lived perennials (0.25), outcrossers (0.27), and late successional taxa (0.30), and lowest diversity in annuals (0.13), selfers (0.12), and early successional taxa (0.17). We found very little total gene diversity in *L. rhynchopetalum* (0.12), even if this species is a long‐lived, perennial and outcrosser (Hedberg, 1964). Very little genetic diversity has also been found in other afroalpine species, possibly due to strong fragmentation and bottlenecks during climatically unfavorable periods (Gizaw et al., 2013; Masao et al., 2013; Wondimu et al., 2014), rendering them particularly vulnerable to further climate change. A meta‐analysis of the impact of habitat fragmentation revealed a substantial decrease in expected heterozygosity, number of alleles, and percentage of polymorphic loci without substantial variation among organisms with different life history traits (Vranckx, Jacquemyn, Muys, & Honnay, 2012). For *L. rhynchopetalum*, it seems therefore likely that further fragmentation into very small areas under future climates will accelerate decrease in its genetic diversity and possibility for survival. Species which are expected to lose 80% of their populations within 100 years are proposed to be listed as critically endangered by the IUCN (IUCN, 2001). Thus, inferred from habitat loss alone, climate change will seriously threaten the survival of *L. rhynchopetalum*. In addition, mountain species are expected to suffer more from climate‐induced range reductions (Frei et al., 2010; Lenoir et al., 2008; Telwala et al., 2013; Thuiller et al., 2005) and the associated loss in genetic diversity (Alsos et al., 2012) than species from lower and warmer regions.

### 4.3 Implications from model calibration and sampling design

We found that the different statistical models and the different complexity levels for calibrating the models revealed very similar results. This is unanticipated, as previous studies have shown that the choice of statistical models generates high uncertainty in projected future ranges of suitable habitats (Buisson, Thuiller, Casajus, Lek, & Grenouillet, 2010). In addition, our tests against independent data reveal that the more complex parameterization schemes did not improve, but rather deteriorate the model accuracies. This is in line with the results of Randin et al. (2006), who also found the simpler GLM models to outperform GAM models when transferred between Eastern Austria and Western Switzerland.

Merow et al. (2014) suggest using simple modeling schemes for data sets that are small and lack sound statistical designs. We believe that our data were well sampled and larger than minimal and thus would allow for more complex model calibration. We restricted our study area to highlands (>2,700 m), thus resulting in a relatively small area. *L. rhynchopetalum* is currently ubiquitous across Ethiopian alpine areas (Fetene et al., 1998; Thulin, 2006) and seems to be in equilibrium with its niche requirements. The sampling design of presence and absence observations covers the most important ecological gradients, which are latitude, altitudes, and aspect. This may also have led to few false absences and little noise in the data set which in turn potentially minimized the differences in performance among models and model complexity levels. This may show how sampling strategy may be more important than selections of model algorithms and model settings, especially for species that appear to be in equilibrium with their niche requirements.

Although the different statistical models and the two different complexity levels for calibrating these models produced very similar results under current climate, the different cut thresholds to produce binary presence–absence predictions added considerable uncertainties to the predictions. Notably, the PT was quite different and mapped a much smaller area as suitable than the other two methods. Using the prevalence of the data used for model building as a threshold is not a common practice but it considered as one of the most robust approaches (Liu, Berry, Dawson, & Pearson, 2005). Although it is recommended to be used for data sets in which the presence–absence data are systematically collected (Halvorsen, 2013; Liu et al., 2005), it is still considered useful in other data sets (Liu et al., 2005). The effect of this model step for analyzing projected gain and loss in suitable habitat is not well studied to date and requires further exploration.

Relying on different algorithms for modeling and combining uncertainty sources in an ensemble map is becoming a common practice (Buisson et al., 2010). Here, we produced multiple maps not only by combining different algorithms of different statistical properties but also models of different complexity levels and their binary maps produced using different threshold criteria, which means we have further enhanced the sources of uncertainties. Increasing uncertainty sources by fitting models of different complexity levels and using more than one threshold criterion per model has not been common practice so far. Although it adds more uncertainty sources, the consensus in predicting suitable habitat among these multiple maps gives more confidence for management and conservation practices, especially in delineating priority areas for conservation purposes and reintroductions of endangered species. It also enhances reliability of model results.

## Conflict of Interest

None declared.
