# Supplementary material for: Good‐bye to tropical alpine plant giants under warmer climates? Loss of range and genetic diversity in Lobelia rhynchopetalum
Source: Ecol Evol. 2016 Nov 25;6(24):8931–41. doi: 10.1002/ece3.2603 (PMC5192889; doi:10.1002/ece3.2603)
Supplement: Supplementary file 1 [file ECE3-6-8931-s001.docx]

Supplementary I: supplementary figures and tables


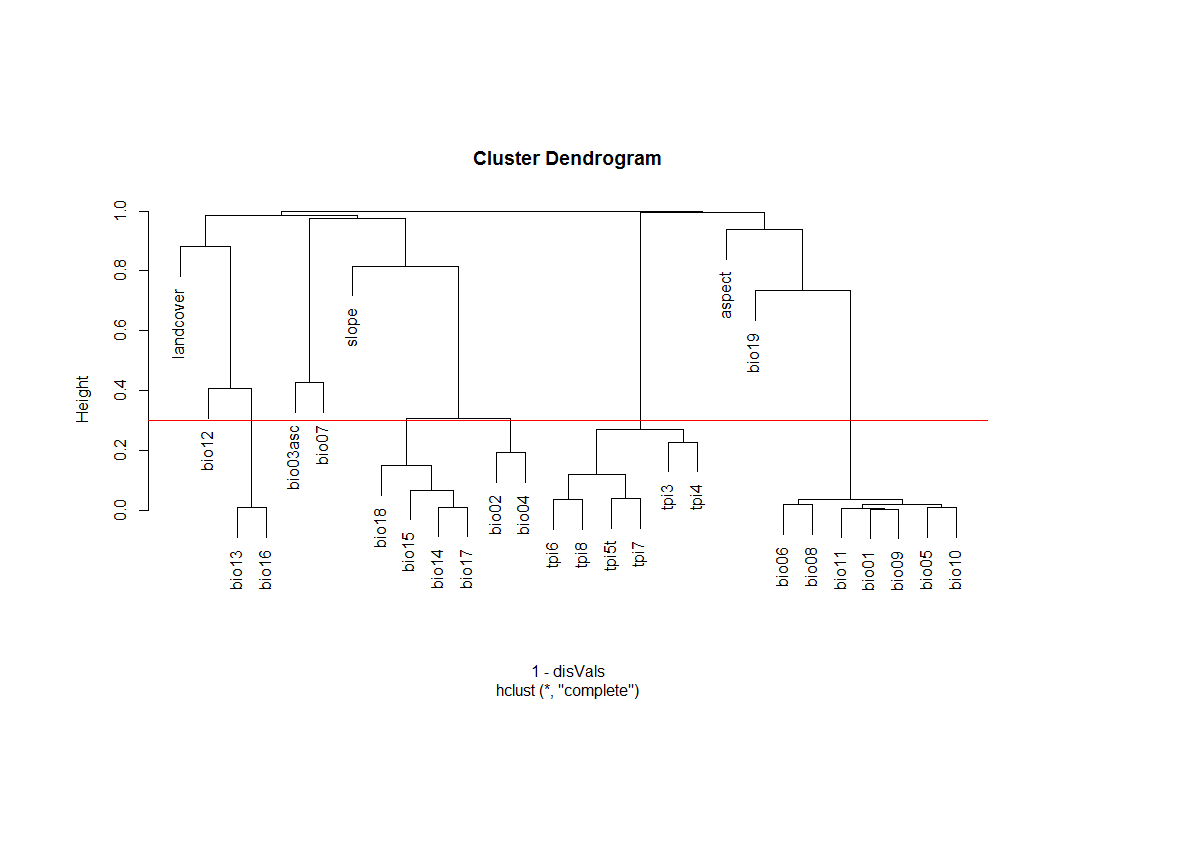


Figure S1: Pairwise Pearson correlation of the predictor variables at locations of training and evaluation data sets. The vertical line indicates a correlation of 0.7 (dissimilarity of 0.3) between variables**.**


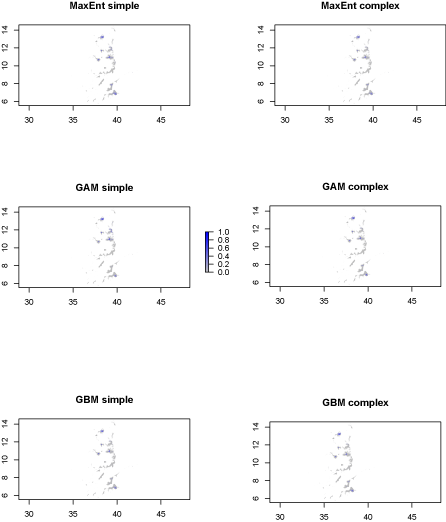


Figure S2: Predicted distribution of the three models calibrated with two complexity levels under current climate.


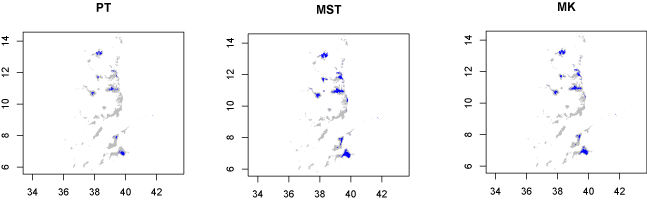


Figure S3: Binary maps of the MaxEnt simple model at different probability thresholds showing suitable habitats in blue and unsuitable habitats in gray under current climates. The probability thresholds are set as follows: PT = prevalence threshold, MST = maximum sum threshold (maximum sum of sensitivity and specificity) and MK = maximized Kappa.


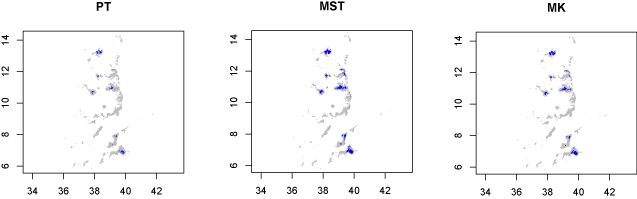


Figure S4: Binary maps of the MaxEnt complex model at different probability thresholds showing suitable habitats in blue and unsuitable habitats in gray under current climates. The probability thresholds are set as follows: PT = prevalence threshold, MST = maximum sum threshold (maximum sum of sensitivity and specificity) and MK = maximized Kappa.

**
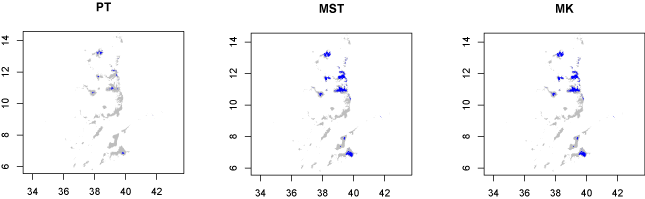
**

Figure S5: Binary maps of the GAM simple model at different probability thresholds showing suitable habitats in blue and unsuitable habitats in gray. The probability thresholds are set as follows: PT = prevalence threshold, MST = maximum sum threshold (maximum sum of sensitivity and specificity) and MK = maximized Kappa.


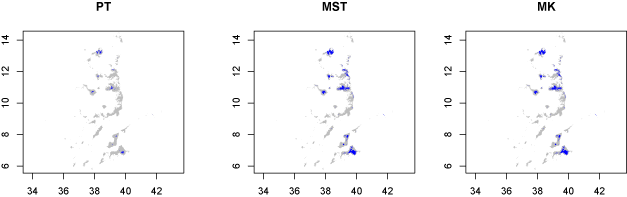


Figure S6: Binary maps of the GAM complex model at different probability thresholds showing suitable habitats in blue and unsuitable habitats in gray. The probability thresholds are set as follows: PT = prevalence threshold, MST = maximum sum threshold (maximum sum of sensitivity and specificity) and MK = maximized Kappa.

**
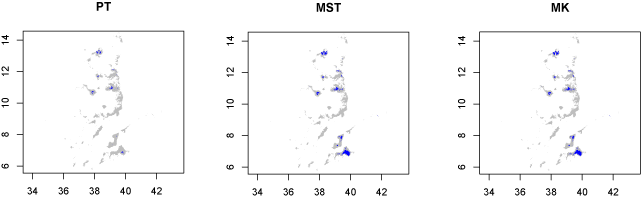
**

Figure S7: Binary maps of the GBM simple model at different probability thresholds showing suitable habitats in blue and unsuitable habitats in gray The probability thresholds are set as follows: PT = prevalence threshold, MST = maximum sum threshold (maximum sum of sensitivity and specificity) and MK = maximized Kappa.

**
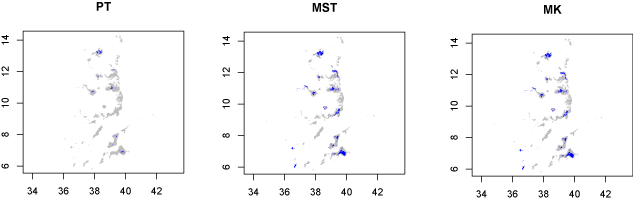
**

Figure S8: Binary maps of the GBM complex model at different probability thresholds showing suitable habitats in blue and unsuitable habitats in gray. The probability thresholds are set as follows: PT = prevalence threshold, MST = maximum sum threshold (maximum sum of sensitivity and specificity) and MK = maximized Kappa.


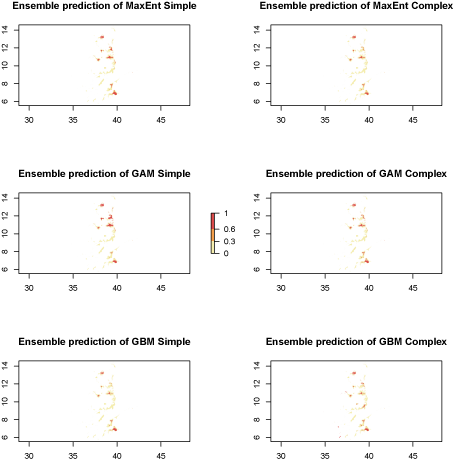


Figure S9: Mean value of predicted suitable habitat (among three thresholds and two model complexity levels): 0.0–0.3 unsuitable habitat with high certainty, 0.3–0.6 uncertain, > 0.6 suitable with high certainty.


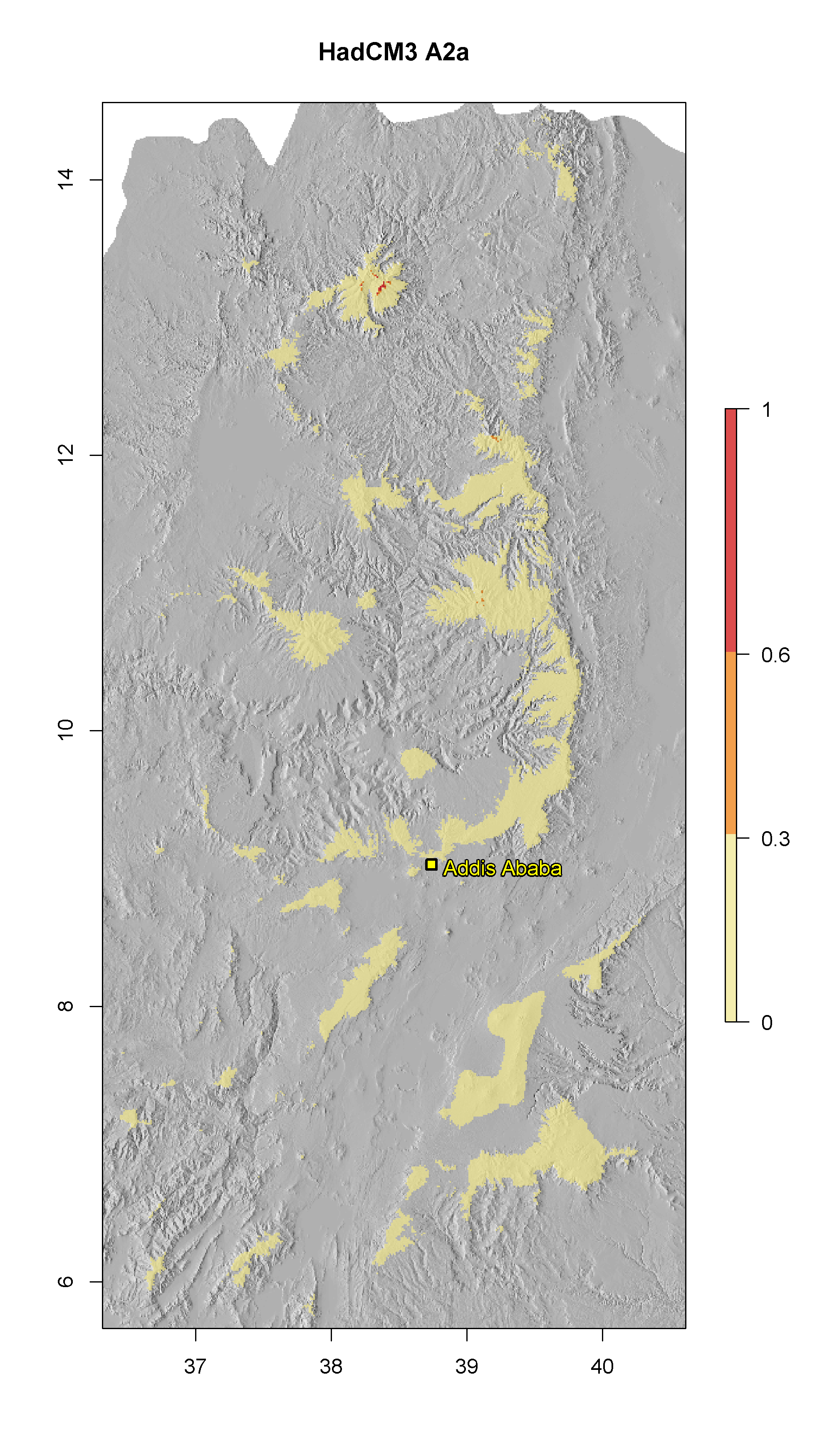


Figure S10: Projected habitat suitability of *L. rhynchopetalum* from overlaying 18 binary presence-absence maps (using three statistical methods calibrated with two complexity levels and cut to presence/absence using three probability thresholds) for future climate scenario HadCM3 A2a, 2080. The mean of classified suitable habitat is meant to represent the following three levels: 0.0-0.3 = habitat is unsuitable with high certainty; 0.3–0.6 = habitat suitability is uncertain, > 0.6 = habitat is suitable with high certainty.


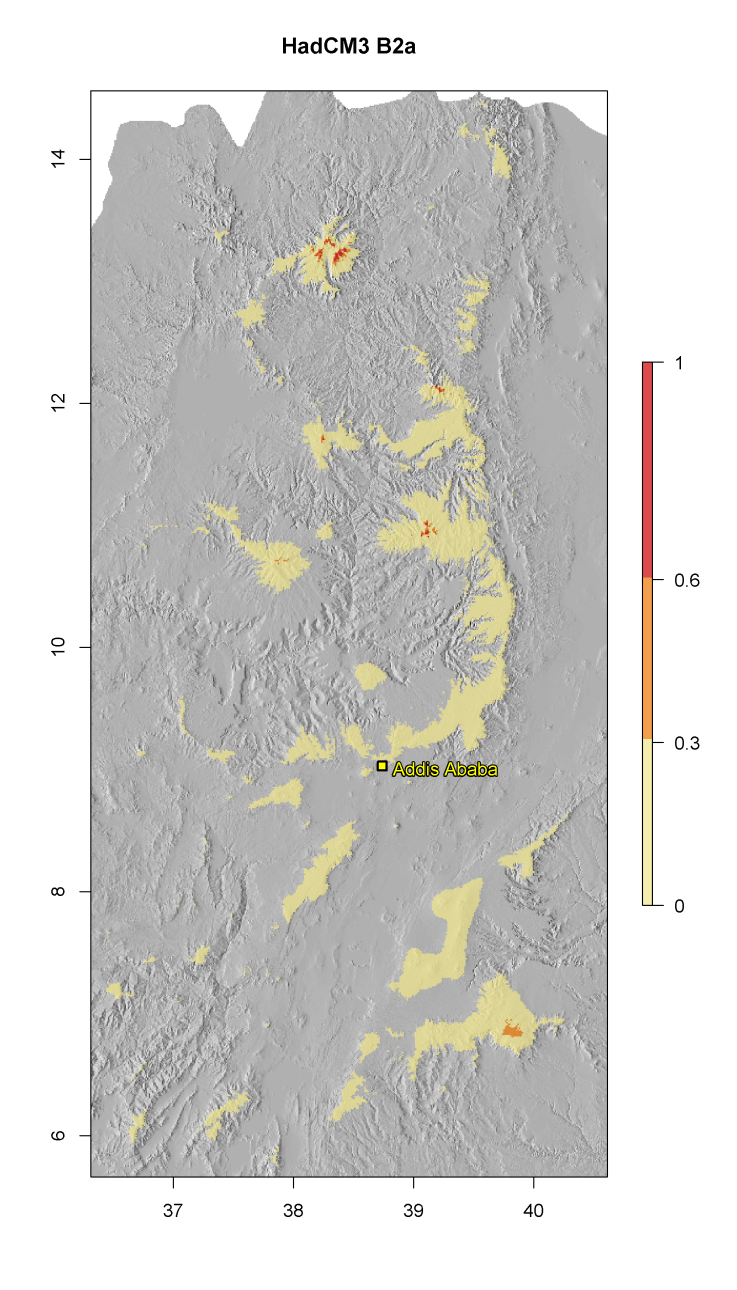


Figure S11: Projected habitat suitability of *L. rhynchopetalum* from overlaying 18 binary presence-absence maps (using three statistical methods calibrated with two complexity levels and cut to presence/absence using three probability thresholds) for future climate scenario HadCM3 B2a, 2080 The mean of classified suitable habitat is meant to represent the following three levels: 0.0-0.3 = habitat is unsuitable with high certainty; 0.3–0.6 = habitat suitability is uncertain, > 0.6 = habitat is suitable with high certainty.


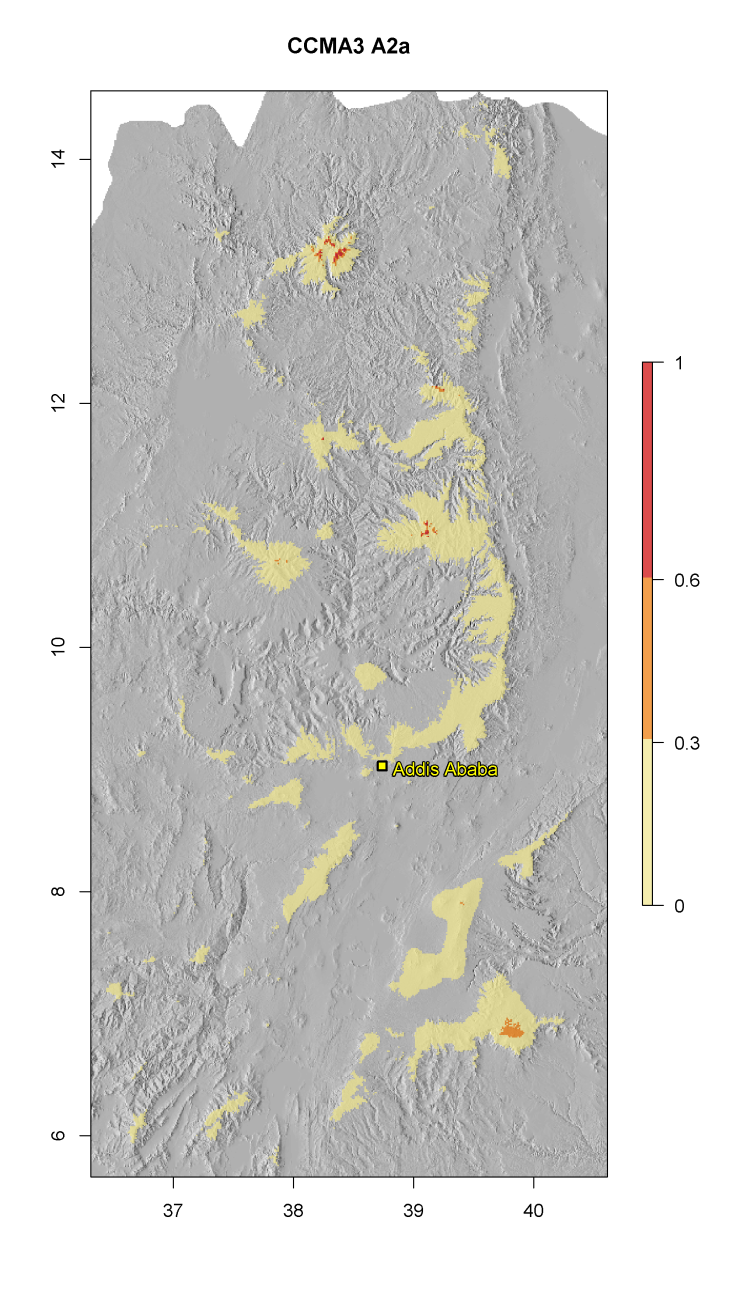


Figure S12: Projected habitat suitability of *L. rhynchopetalum* from overlaying 18 binary presence-absence maps (using three statistical methods calibrated with two complexity levels and cut to presence/absence using three probability thresholds) for future climate scenario CCMA3 A2a, 2080. The mean of classified suitable habitat is meant to represent the following three levels: 0.0-0.3 = habitat is unsuitable with high certainty; 0.3–0.6 = habitat suitability is uncertain, > 0.6 = habitat is suitable with high certainty.


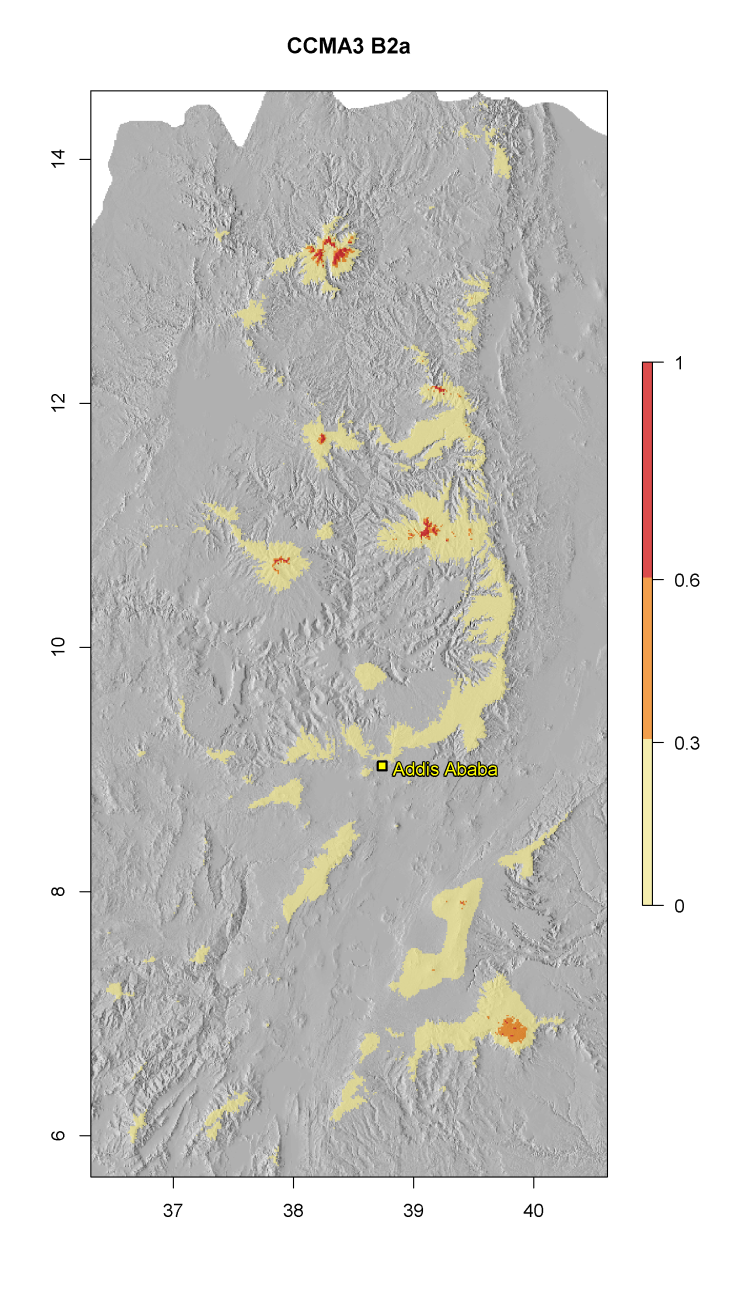


Figure S13: Projected habitat suitability of *L. rhynchopetalum* from overlaying 18 binary presence-absence maps (using three statistical methods calibrated with two complexity levels and cut to presence/absence using three probability thresholds) for future climate scenario CCMA3 B2a, 20800. The mean of classified suitable habitat is meant to represent the following three levels: 0.0-0.3 = habitat is unsuitable with high certainty; 0.3–0.6 = habitat suitability is uncertain, > 0.6 = habitat is suitable with high certainty.


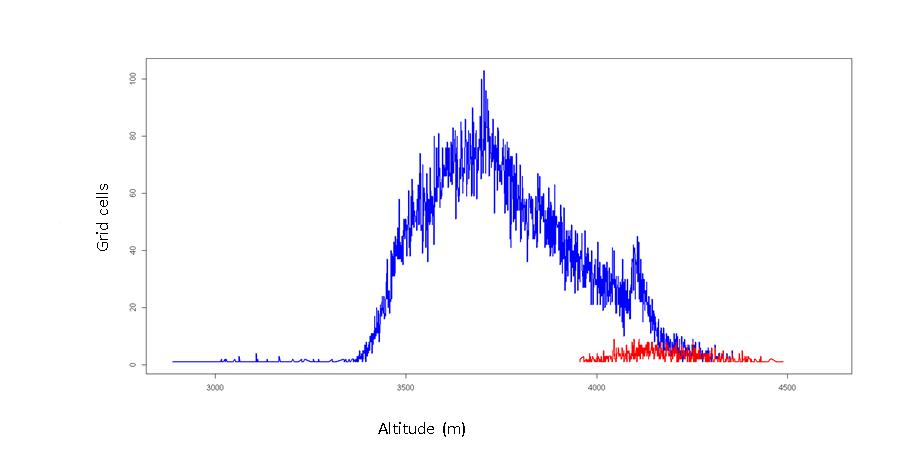


Figure S14: Number of grid cells (270 m x 270 m) projected to be suitable with high certainty along the elevational gradient under current (blue) and future (red) climate.


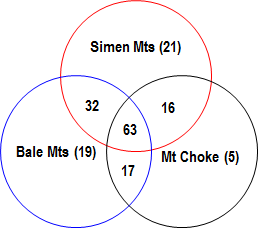


Figure S15: Number of private (in parentheses) and shared AFLP markers in the three mountains systems analyzed.


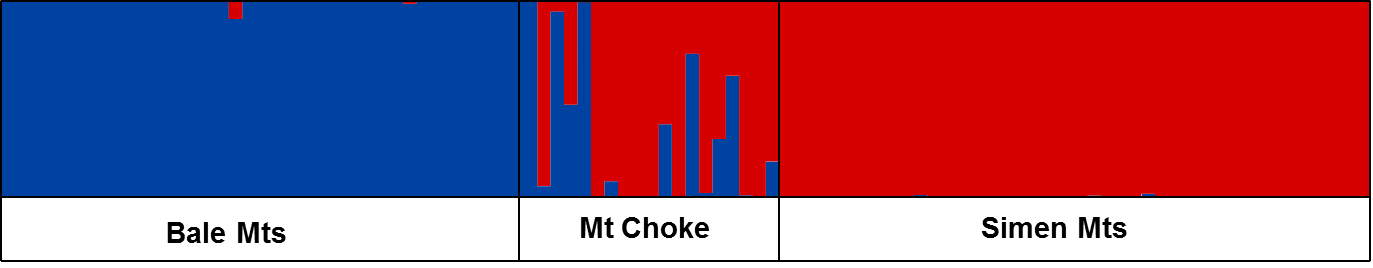


Figure S16: Genetic structuring in *L. rhynchopetalum* in the three mountain systems inferred from STRUCTURE analyses.

Table S1: Number of transects and plots investigated in the field, and number of plots where *L. rhyncopetalum* was recorded as present.

| Mountain system | No of transects | No of plots | No of plots with *L. rhynchopetalum* |
| --- | --- | --- | --- |
| Bale Mts | 9 | 116 | 9 |
| Mt Choke | 7 | 41 | 24 |
| Simen Mts | 8 | 61 | 54 |
| Total | 24 | 218 | 87 |

Table S2: Performance of the three model algorithms evaluated against independent presence-absence data.

| Model | Test AUC |
| --- | --- |
| MaxEnt simple | 0.967 |
| MaxEnt complex | 0.957 |
| GAM simple | 0.948 |
| GAM complex | 0.945 |
| GBM simple | 0.973 |
| GBM complex | 0.970 |
|  |  |

Table S3: proportion (%) of the study area that is predicted as non-suitable with high certainty, uncertain and suitable with high certainty under different gas emission scenarios and in the overall ensemble maps of current and future habitat ranges.

|  | Current | HadCM3 A2a | HadCM3 B2a | CCMA3 A2a | CCMA3 B2a | Future |
| --- | --- | --- | --- | --- | --- | --- |
| Unsuitable | 84.82 | 99.78 | 98.59 | 98.61 | 96.49 | 98.66 |
| Uncertain | 7.56 | 0.15 | 1.07 | 1.11 | 2.56 | 1.07 |
| Suitable | 7.62 | 0.07 | 0.34 | 0.28 | 0.96 | 0.27 |

Table S4: Analyses of molecular variance (AMOVAs) based on AFLP data for 21 populations of *L. rhynchopetalum* from the three mountain systems (Bale Mts, Mt Choke and Simen Mts). All P - values were significant (P < 0.0001; estimated in a permutation test of 1000 permutations).

| Source of variation | d.f. | Sum of squares | Variance components | % of total variance |
| --- | --- | --- | --- | --- |
| Among mountain systems | 2 | 127.56 | 1.54 | 12.22 |
| Among populations within mountain systems | 18 | 264.31 | 0.96 | 7.65 |
| Within populations | 80 | 805.15 | 10.06 | 80.13 |
| Total | 100 | 1197.03 | 12.56 |  |

Table S5: Genetic diversity and rarity in populations of *L. rhynchopetalum.*

| Mountain system | Population number | Corema ID | Locality | Latitude (N) | Longitude (E) | Altitude  (m) | *n* | *D* | *P* (%) | DW |
| --- | --- | --- | --- | --- | --- | --- | --- | --- | --- | --- |
| Bale Mts | ET0519 | O-DP-31268 - 31271 | Bale Mts: Garba Guracha | 6.8687 | 39.8822 | 4020 | 4 | 0.133 | 24.28 | 1.82 |
|  | ET0538 | O-DP-44380,  O-DP-45053 - 45055 | - | - | - | - | 4 | 0.106 | 19.65 | 1.48 |
|  | ET0633 | O-DP-31666,  O-DP-45128 - 45131 | Bale Mts: Sanetti Plateau | 6.8550 | 39.8780 | 4140 | 5 | 0.090 | 19.65 | 1.21 |
|  | ET0706 | O-DP-31849 - 31853 | Bale Mts: Garba Guracha | 6.8793 | 39.8690 | 3960 | 5 | 0.126 | 26.01 | 2.39 |
|  | ET0765 | O-DP-32065 - 32069 | Bale Mts: Garba Guracha | 6.8703 | 39.8678 | 4105 | 5 | 0.089 | 18.50 | 1.77 |
|  | ET0811 | O-DP-42114 - 42115,  O-DP-32256 - 32258 | Bale Mts: Sanetti, Konten | 6.8448 | 39.8805 | 4105 | 5 | 0.098 | 20.23 | 1.36 |
|  | ET0933 | O-DP-32785,  O-DP-42026 - 42029 | Bale Mts: Angaso | 6.8822 | 39.8883 | 3950 | 5 | 0.139 | 29.48 | 2.36 |
|  | ET1029 | O-DP-33134 - 33139 | Bale Mts: Angaso | 6.8931 | 39.8974 | 3875 | 5 | 0.131 | 26.59 | 1.51 |
| Mt Choke | ET1331 | O-DP-33611 - 33612,  O-DP-33614 - 33615 | Mt Choke | 10.6420 | 37.8357 | 3830 | 4 | 0.120 | 22.54 | 1.96 |
|  | ET1354 | O-DP-33726 - 33730 | Mt Choke | 10.6560 | 37.8257 | 3815 | 5 | 0.116 | 24.86 | 1.98 |
|  | ET1378 | O-DP-33846 - 33850 | Mt Choke | 10.6382 | 37.8392 | 3830 | 5 | 0.108 | 23.70 | 1.34 |
|  | ET1382 | O-DP-33866 - 33870 | Mt Choke | 10.6575 | 37.8220 | 3840 | 5 | 0.120 | 26.01 | 1.78 |
| Simen Mts | ET0122 | O-DP-29728 - 29732 | Simen Mts: Saha | 13.2827 | 38.1108 | 3700 | 5 | 0.104 | 20.81 | 1.51 |
|  | ET0157 | O-DP-29850 - 29854 | Simen Mts: Saha | 13.2853 | 38.1184 | 3700 | 5 | 0.092 | 19.65 | 1.37 |
|  | ET0324 | O-DP-30493 - 30497 | Simen Mts: Bwahit | 13.2514 | 38.2023 | 4040 | 5 | 0.131 | 30.64 | 1.69 |
|  | ET0325 | O-DP-30498 - 30502 | Simen Mts: Chenek | 13.2600 | 38.2000 | 3725 | 5 | 0.099 | 21.39 | 1.09 |
|  | ET0432 | O-DP-30907 - 30911 | Simen Mts: Silki | 13.3491 | 38.2625 | 3945 | 5 | 0.101 | 20.23 | 1.26 |
|  | ET0454 | O-DP-30998 - 31000,  O-DP-31002 | Simen Mts: Silki | 13.3333 | 38.2333 | 3430 | 4 | 0.137 | 26.59 | 1.50 |
|  | ET0462 | O-DP-31032 - 31036 | Simen Mts: Silki | 13.3285 | 38.2430 | 3680 | 5 | 0.142 | 31.79 | 1.91 |
|  | ET0558 | O-DP-31440 - 31444 | Simen Mts: Silki | 13.3285 | 38.2409 | 3655 | 5 | 0.116 | 24.86 | 2.26 |
|  | ET0621 | O-DP-42130 - 42134 | Simen Mts: Silki | 13.3271 | 38.2425 | 3690 | 5 | 0.154 | 32.37 | 2.40 |

*n* - number of individuals successfully analyzed, *P*(%) - percentage of polymorphic loci, DW - frequency-down-weighted marker value as a measure of genetic rarity; *D* - Nei’s gene diversity

Table S6: Estimated gene diversity and proportion of markers lost as a consequence of 76-95% habitat range loss, which corresponds to 5-1 remaining random populations. The proportion of markers lost is calculated after subtracting the average number of markers per individual, following Alsos *et al*. (2012).

|  |  | Expected gene diversity | | | Expected proportion of markers lost (%) | | |
| --- | --- | --- | --- | --- | --- | --- | --- |
| No of remaining populations | Area lost (%) | Mean | 95 CI lower | 95 CI upper | Mean | 95 CI lower | 95 CI upper |
| 5 | 76.19 | 0.14 | 0.12 | 0.15 | 37.37 | 28.44 | 47.71 |
| 4 | 80.95 | 0.13 | 0.12 | 0.15 | 44.92 | 33.79 | 55.40 |
| 3 | 85.71 | 0.13 | 0.11 | 0.15 | 54.67 | 43.85 | 63.85 |
| 2 | 90.48 | 0.13 | 0.10 | 0.16 | 65.81 | 54.62 | 75.40 |
| 1 | 95.24 | 0.12 | 0.09 | 0.14 | 81.61 | 74.58 | 86.92 |

Table S7: Estimated gene diversity and proportion of markers lost as a consequence of 76-95% habitat range loss, which corresponds to 5-1 remaining random populations (if several populations remained, they were constrained to be on different mountains). The proportion of markers lost is calculated after subtracting the average number of markers per individual, following Alsos et al. 2012.

|  |  | Expected gene diversity | | | Expected proportion of markers lost (%) | | |
| --- | --- | --- | --- | --- | --- | --- | --- |
| No of remaining populations | Area lost (%) | Mean | 95 CI lower | 95 CI upper | Mean | 95 CI lower | 95 CI upper |
| 5 | 76.19 | 0.14 | 0.12 | 0.15 | 37.37 | 28.44 | 47.71 |
| 4 | 80.95 | 0.13 | 0.12 | 0.15 | 44.92 | 33.79 | 55.40 |
| 3 | 85.71 | 0.13 | 0.11 | 0.15 | 54.67 | 43.85 | 63.85 |
| 2 | 90.48 | 0.13 | 0.10 | 0.16 | 65.81 | 54.62 | 75.40 |
| 1 | 95.24 | 0.12 | 0.09 | 0.14 | 81.61 | 74.58 | 86.92 |

**Reference**

Alsos, I.G., Ehrich, D., Thuiller, W., Eidesen, P.B., Tribsch, A., Schonswetter, P., Lagaye, C., Taberlet, P., Brochmann, C. (2012) Genetic consequences of climate change for northern plants. Proceedings. Biological sciences/The Royal Society **279**, 2042-2051.
