# Supplementary material for: Good‐bye to tropical alpine plant giants under warmer climates? Loss of range and genetic diversity in Lobelia rhynchopetalum
Source: Ecol Evol. 2016 Nov 25;6(24):8931–41. doi: 10.1002/ece3.2603 (PMC5192889; doi:10.1002/ece3.2603)
Supplement: Supplementary file 2 [file ECE3-6-8931-s002.docx]

**Appendix S1: Collection and analysis of genetic data**

Field sampling, DNA extraction and AFLP fingerprinting

Fresh young leaf samples were collected from three mountain systems in the Ethiopian Highlands: Bale Mts, Mt Choke and Simen Mts. Five plants, taken to represent a single population, were collected from each of four randomly selected plots (100 m x 100 m) in each mountain system. Leaf samples were dried in silica gel, and voucher specimens of the five sampled individuals were pressed and deposited in the Natural History Museum, University of Oslo, Norway (1 voucher) and the remaining in National Herbarium of Ethiopia, Addis Ababa University. A total of 102 individual plants representing 21 populations were sampled.

Total genomic DNA was extracted from the dried leaves using MoleStrips^TM^ DNA Plant Kit with an automated GeneMole^®^ robot following the manufacturer’s instructions (Qiagen Nordic) with some modifications. Leaf tissue was mechanically ground in 2.0 mL tubes with two tungsten carbide beads for 2 min at 15 Hz in a mixer mill (MM301, Retsch GmbH & Co., Haan, Germany). Three hundred µL of lysis buffer was added to the crushed material, vortexed, spinned for 20 sec at 3400 Hz, incubated for 15 min at 65°C, and spinned at 14000 Hz for 3 min. Two hundred micro-litres of the lysate were transferred into new tubes and loaded to the GeneMole^®^ robot.

AFLP analysis was performed to assess genome-wide genetic variation following Gaudeul et al. (2000) except that the PCR reaction volumes were reduced by 50% and pre-selective PCR products were diluted ten times. Thirty primer combinations were tested using two samples from different mountains and 12 of them resulting in high reproducibility and many scorable polymorphic loci were further tested using eight samples. The final AFLP analyses were carried out using the three most promising primer combinations (*EcoRI* - AGA - (6FAM) - *Msel* – CAC, *EcoRI* - AGG - (VIC) - *Msel* – CTG and *EcoRI* -ACC - (NED) - *Msel* - CTG). For each sample, 2 μL 6FAM, 2 μL VIC and 3 μL NED labelled selective PCR products were diluted in 14 μL distilled water. A total volume of 3.5 μL of the diluted reaction was mixed with 11.7 μL formamide and 0.3 μL GeneScan^TM^ 500 ROX^TM^ internal-lane size standards and denatured at 95°C for 5 min, and cooled on ice before running on an ABI 3100 sequencer (Applied Biosystems). The raw data were analyzed using ABI prism GeneScan version 3.7 (Applied Biosystems) and imported for scoring into GeneMapper version 4.0 (Applied Biosystems). AFLP fragments in the size range 50–500 base pairs (bp) were automatically scored as present (1) or absent (0). The error rate was assessed from 16 randomly selected duplicates representing 15.7% of the total sample size (Bonin *et al.*, 2004). Twelve of the duplicates were re-extracted from new leaf material while the same DNA extracts were used for the remaining four.

Data analysis

Unique markers and markers with low reproducibility were removed. Based on 16 replicates, the error rate was 2.7%.

To quantify genetic diversity, we estimated the proportion of polymorphic markers (*P*) and Nei’s gene diversity (*D*; estimated as the average proportion of pairwise differences among genotypes; Kosman, 2003) using the R-script implemented in AFLPdat (Ehrich, 2006). Genetic rarity was estimated as frequency-down-weighted marker values (*Ra*) according to Schönswetter &Tribsch (2005) using AFLPdat (Ehrich, 2006). In addition, we counted the number of AFLP markers that were private (endemic) to each mountain system and the number shared among them.

Pairwise genetic similarity among AFLP multilocus genotypes was calculated using Dice’s coefficient of similarity and visualized by Principal Coordinate Analysis (PCoA) using NTSYSpc version 2.11a (Rohlf, 2000). We performed analyses of molecular variance (AMOVAs) to investigate the partitioning of genetic variation at different hierarchical levels using ARLEQUIN version 3.5 (Excoffier & Lischer, 2010). Population structure was further examined using Bayesian model-based clustering methods implemented in STRUCTURE version. 2.3.3 (Pritchard *et al.*, 2000). The recessive allele model was used to account for the dominant nature of AFLP data (Falush *et al*., 2007). We used admixture model with correlated allele frequencies and run ten replicates for each *K* ranging from 1 to 7 using a burn-in period of 200000 and 1000000 iterations. The optimal value of *K* was inferred based on the estimated log likelihoods, *L*(*K*), and the stability of assignment patterns across runs. In addition, the rate of change in the probability between successive *K*, Δ*K*, was taken into account (Evanno *et al*., 2005). We used the R-script STRUCTURE-SUM (Ehrich, 2007) to summarize the results and to infer the optimal value of *K* based on these criteria.

**References**

Ehrich, D. (2006) AFLPdat: a collection of R functions for convenient handling of AFLP data. *Moleuclar Ecology Notes,* **6**:603–604.

Ehrich, D., Gaudeul, M., Assefa, A., Koch, M.A., Mummenhoff, K., Nemomissa, S., Brochmann, C. (2007) Genetic consequences of Pleistocene range shifts: contrast between the Arctic, the Alps and the East African mountains. *Moleuclar Ecology*, **16**:2542–2559.

Evanno, G., Regnaut, S., Goudet, J. (2005) Detecting the number of clusters of individuals using the software STRUCTURE: a simulation study. *Moleuclar Ecology*, **14**:2611–2620.

Excoffier, L., & Lischer, H. E. L. (2010) Arlequin suite ver. 3.5: a new series of programs to perform population genetics analyses under Linux and Windows. *Moleuclar Ecology Resources*, **10**:564–567.

Falush, D., Stephens, M., Pritchard, J. (2007) Inference of population structure using multilocus genotype data: dominant markers and null allele. *Moleuclar Ecology Notes*, **7**:574–578.

Kosman, E. (2003) Nei’s gene diversity and the index of average differences are identical measures of diversity within populations. *Plant Pathology,* **52**:533–535

Pritchard, J. K., Stephens, M., Donnelly, P. (2000) Inference of population structure using multilocus genotype data. *Genetics*, **155**:945–959.

Rohlf, F. (2000) NTSYSpc: Numerical Taxonomy and Multivariate Analysis System. Version 2.11a. Setauket (NY): Exeter Software

Schönswetter, P. & Tribsch, A. (2005) Vicariance and dispersal in the alpine perennial *Bupleurum stellatum* L. (Apiaceae). *Taxon*, **54**, 725-732.
